# Relationships between drug activity in NCI preclinical in vitro and in vivo models and early clinical trials

**DOI:** 10.1054/bjoc.2001.1796

**Published:** 2001-05

**Authors:** J I Johnson, S Decker, D Zaharevitz, L V Rubinstein, J M Venditti, S Schepartz, S Kalyandrug, M Christian, S Arbuck, M Hollingshead, E A Sausville

**Affiliations:** Developmental Therapeutics Program,Cancer Therapy Evaluation Program, Division of Cancer Treatment and Diagnosis, National Cancer Institute, Bethesda, MD 20892, andSAIC-Frederick, Frederick, MD 21701

**Keywords:** anticancer drug discovery, in vitro-to-in vivo correlations, clinical trials

## Abstract

An analysis of the activity of compounds tested in pre-clinical in vivo and in vitro assays by the National Cancer Institute's Developmental Therapeutics Program was performed. For 39 agents with both xenograft data and Phase II clinical trials results available, in vivo activity in a particular histology in a tumour model did not closely correlate with activity in the same human cancer histology, casting doubt on the correspondence of the pre-clinical models to clinical results. However, for compounds with in vivo activity in at least one-third of tested xenograft models, there was correlation with ultimate activity in at least some Phase II trials. Thus, an efficient means of predicting activity in vivo models remains desirable for compounds with anti-proliferative activity in vitro. For 564 compounds tested in the hollow fibre assay which were also tested against in vivo tumour models, the likelihood of finding xenograft activity in at least one-third of the in vivo models tested rose with increasing intraperitoneal hollow fibre activity, from 8% for all compounds tested to 20% in agents with evidence of response in more than 6 intraperitoneal fibres (*P*< 0.0001). Intraperitoneal hollow fibre activity was also found to be a better predictor of xenograft activity than either subcutaneous hollow fibre activity or intraperitoneal plus subcutaneous activity combined. Since hollow fibre activity was a useful indicator of potential in vivo response, correlates with hollow fibre activity were examined for 2304 compounds tested in both the NCI 60 cell line in vitro cancer drug screen and hollow fibre assay. A positive correlation was found for histologic selectivity between in vitro and hollow fibre responses. The most striking correlation was between potency in the 60 cell line screen and hollow fibre activity; 56% of compounds with mean 50% growth inhibition below 10^–7.5^ M were active in more than 6 intraperitoneal fibres whereas only 4% of compounds with a potency of 10^–4^ M achieved the same level of hollow fibre activity (*P*< 0.0001). Structural parameters of the drugs analysed included compound molecular weight and hydrogen-bonding factors, both of which were found to be predictive of hollow fibre activity. © 2001 Cancer Research Campaign www.bjcancer.com


*British Journal of Cancer* (2001) **84**(10), 1424–1431
© 2001 Cancer Research Campaign
doi: 10.1054/ bjoc.2001.1796, available online at http://www.idealibrary.com on IDEAL

http://www.bjcancer.com

### Relationships between drug activity in NCI preclinical in vitro and in vivo models and early clinical trials

**JI Johnson,[1] S Decker,[1] D Zaharevitz,[1] LV Rubinstein,[2] JM Venditti,[3] S Schepartz,[3] S Kalyandrug,[2] M Christian,[2] S Arbuck,[2] M Hollingshead[1] and EA Sausville[1]**

[1]Developmental Therapeutics Program, [2]Cancer Therapy Evaluation Program, Division of Cancer Treatment and Diagnosis, National Cancer Institute, Bethesda, MD 20892, and [3]SAIC-Frederick, Frederick, MD 21701

**Summary** An analysis of the activity of compounds tested in pre-clinical in vivo and in vitro assays by the National Cancer Institute's Developmental Therapeutics Program was performed. For 39 agents with both xenograft data and Phase II clinical trials results available, in vivo activity in a particular histology in a tumour model did not closely correlate with activity in the same human cancer histology, casting doubt on the correspondence of the pre-clinical models to clinical results. However, for compounds with in vivo activity in at least one-third of tested xenograft models, there was correlation with ultimate activity in at least some Phase II trials. Thus, an efficient means of predicting activity in vivo models remains desirable for compounds with anti-proliferative activity in vitro. For 564 compounds tested in the hollow fibre assay which were also tested against in vivo tumour models, the likelihood of finding xenograft activity in at least one-third of the in vivo models tested rose with increasing intraperitoneal hollow fibre activity, from 8% for all compounds tested to 20% in agents with evidence of response in more than 6 intraperitoneal fibres ($P < 0.0001$). Intraperitoneal hollow fibre activity was also found to be a better predictor of xenograft activity than either subcutaneous hollow fibre activity or intraperitoneal plus subcutaneous activity combined. Since hollow fibre activity was a useful indicator of potential in vivo response, correlates with hollow fibre activity were examined for 2304 compounds tested in both the NCI 60 cell line in vitro cancer drug screen and hollow fibre assay. A positive correlation was found for histologic selectivity between in vitro and hollow fibre responses. The most striking correlation was between potency in the 60 cell line screen and hollow fibre activity; 56% of compounds with mean 50% growth inhibition below $10^{-7.5}$ M were active in more than 6 intraperitoneal fibres whereas only 4% of compounds with a potency of $10^{-4}$ M achieved the same level of hollow fibre activity ($P < 0.0001$). Structural parameters of the drugs analysed included compound molecular weight and hydrogen-bonding factors, both of which were found to be predictive of hollow fibre activity. © 2001 Cancer Research Campaign http://www.bjcancer.com

**Keywords**: anticancer drug discovery; in vitro-to-in vivo correlations; clinical trials

Since its inception in 1955, the National Cancer Institute's (NCI) Developmental Therapeutics Program (DTP) has utilized various experimental screening models to select agents for evaluation as clinical candidates. The philosophical position from which this endeavour proceeded was that elucidation of empirically defined anti-tumor activity in a model would translate into some likelihood of activity in human cancer. The choice of specific screening models was based primarily on response of the models to agents already identified as clinically active (Gellhorn and Hirschberg, 1955; Zubrod et al, 1966). Initially, 3 transplanted rodent models were used: Sarcoma 180, Carcinoma 755 and Leukaemia L1210. The spectrum of models was then broadened, retaining L1210, which was discerned to be the most predictive of clinical activity, and adding a series of transplanted rodent models. This scheme was replaced in 1975 by the murine P388 leukaemia model, which was utilized as a pre-screen and followed by a panel of tumours. This panel first included only rodent tumours but was later enhanced to include human tumour xenografts (Venditti, 1981; Venditti et al, 1984). The human tumour xenografts were employed with the intent of their serving as potentially better predictors of clinical activity against solid human tumours.

In early 1990, the P388 pre-screen was replaced by an in vitro human tumour cell line assay comprised of 60 different cell types (Alley et al, 1988; Monks et al, 1991; Paull et al, 1995). Agents selected on the basis of potency, selective activity against a particular disease category, and/or differential activity against a few specific cell lines were then evaluated against a small number of sensitive human tumours in the nude mouse xenograft model (Dykes et al, 1992; Plowman et al, 1997) as a basis for selecting compounds for further preclinical development. Owing to the large numbers of molecules emerging from the in vitro screen as candidates for xenograft testing, in 1995 this development path was further modified to include a hollow fibre (HF) assay (Hollingshead et al, 1999), activity in which was a prerequisite for study in classical xenograft models. The HF model, where cells are introduced from tissue culture into semi-permeable fibres in mouse intraperitoneal (i.p.) or subcutaneous (s.c.) space and exposed to test agents, is a rapid and efficient means of selecting compounds with the potential for in vivo activity in conventional xenografts in pilot and 'training' sets of compounds.

This drug screening and development scheme remains an empirical one, as compounds are prioritized for development based on the definition of anti-proliferative in vitro and in vivo responses. Owing to an emerging understanding of the molecular

*Received 10 August 2000*
*Revised 13 February 2001*
*Accepted 19 February 2001*

*Correspondence to:* EA Sausville basis for human cancer, great interest exists in transitioning from an empirical to a potentially more rational, molecular-targeted approach to the discovery and development of novel cancer therapeutics (Sausville and Feigal, 1999). Of particular interest will be the design of models to detect the action of compounds on particular predefined targets. The 'performance features' of compounds that have been evaluated in the 'empirical' development scheme may be of value in serving as a baseline against which newer compounds and models may be compared.

To this end, we present here an experience attempting to correlate activity in the clinic with antecedent activity in preclinical models. In addition, we present NCI's cumulative experience with the performance of the HF assay in relation to in vitro activity and to certain chemical characteristics of the compounds.

### MATERIALS AND METHODS

### Agents used

Data for 39 agents which had completed phase II trials (Table 1) and which had also been evaluated against in vivo tumour models were compiled. 13 of these compounds were 'standard' agents and the remaining 26 were compounds for which NCI INDs were filed between 1980 and 1996.

For an analysis of factors affecting xenograft outcome, 1228 compounds for which both current xenograft data and activity in NCI's 60 cell line in vitro anti-cancer drug screen were available. The compounds were tested in a median of 5 xenograft experiments (range 1–131) and a median of 4 different histologies (range 1–14). In addition, 564 of these had been evaluated in the HF assay. Compounds were selected for xenograft testing at least partially on the basis of performance in the 60 cell line assay and/or the HF assay. Of these, 756 are open, publicly available compounds and 472 are discreet, confidential structures.

A set of 2304 compounds which was evaluated in the 60 cell line assay (1252 open, 1052 discreet) and referred for evaluation and testing in the HF assay was analysed. The compounds represented a wide variety of structural types, and ranged in in vitro potency from $GI_{50}$s (concentration of drug which achieved 50% growth inhibition averaged over all 60 cell lines) of 100 μM to less than 10 nM. Reasons for referring the compounds for HF evaluation included selective activity against cell lines of a particular histologic type, 'non-standard' mechanism of action as determined by the COMPARE pattern recognition algorithm (Paull et al, 1989), as well as compounds which were structurally novel and possibly representing novel chemotypes directed against defined mechanisms (e.g. topoisomerase inhibition). Lists of the non-proprietary compounds in these data sets are available at http://dtp.nci.nih.gov/docs/bjcwebsup.html.

**Table 1** NSC numbers and common names for 39 phase II clinical agents

| NSC Number | Common name |
|---|---|
| 740 | Methotrexate |
| 3053 | Actinomycin-D |
| 3088 | Chlorambucil |
| 8806 | Melphalan |
| 19893 | 5-FU |
| 26271 | Cyclophosphamide |
| 26980 | Mitomycin C |
| 45388 | Dacarbazine |
| 49842 | Vinblastine |
| 105014 | 2-CDA |
| 119875 | Cisplatin |
| 123127 | Adriamycin HCL |
| 125066 | Bleomycin |
| 125973 | Paclitaxel |
| 141633 | Homoharringtonine |
| 172112 | Spiromustine |
| 253272 | Caracemide |
| 264880 | Dihydro-5-azacytidine |
| 267469 | Deoxydoxorubicin |
| 269148 | Menogaril |
| 281272 | Fazarabine |
| 286193 | Tiazofurin |
| 308847 | Amonafide |
| 312887 | Fludarabine phosphate |
| 325319 | Didemnin B |
| 332598 | Rhizoxin |
| 336628 | Merbarone |
| 337766 | Bisantrene |
| 339004 | Chloroquinoxaline sulfonamide |
| 347512 | Flavone acetic acid |
| 349174 | Piroxantrone hydrochloride |
| 352122 | Trimetrexate |
| 356894 | Deoxyspergualin |
| 361456 | Pyrazine diazohydroxide |
| 366140 | Pyrazoloacridine |
| 409962 | BCNU |
| 609699 | Hycamptamine |
| 616348 | CPT-11 (irinotecan) |
| 628503 | Taxotere (docetaxel) |

### Clinical response

Clinical response rate of particular histologic tumour types to the 26 NCI IND agents was reviewed in patients from all completed phase II trials with at least 9 patients; the trials were conducted under NCI sponsorship or otherwise included in Cancer Therapy Evaluation Program records, and were carried out with appropriate ethical committee approval. A 'positive' phase II trial involved an objective 50% reduction in tumour size in at least 20% of patients. For the 13 standard agents, response rates were taken from DeVita (De Vita et al, 1982, 1985, 1989, 1993, 1997). All abstractions were done by professional abstractors from EMMES Corporation (Potomac, MD).

### Preclinical models

Compounds were tested in a variety of xenograft models according to methods described by Dykes and Plowman (Dykes et al, 1992; Plowman et al, 1997). Two levels of response were considered. For 'survival' models, the 2 thresholds were increased life span of 25% or 50%. In other subcutaneous xenograft models, estimation of tumour weight allowed calculation of treated/control weight ratios (T/C), with thresholds of 40% and 10% indicative of a level or degree of activity in this analysis. Agents were in all cases studied at or below the maximum tolerated dose, defined as the greatest quantity of test agent given as an acute dose which a mouse is able to survive for 11 days.

Agents were tested in the HF assay as described by Hollingshead (Hollingshead et al, 1999). A standard panel of 12 tumour cell lines are used for routine HF screening, including non-small cell lung carcinoma lines NCI-H23 and NCI-H522, breast carcinoma lines MDA-MB-231 and MDA-MB-435, colon sarcoma lines SW-620 and COLO 205, melanoma lines LOX and

UACC-62, ovarian carcinoma lines OVCAR-3 and OVCAR-5, and glioma lines U251 and SF-295. The cells, at densities of 2–10 $\times 10^6$ cells $ml,^{-1}$ are flushed into polyvinylidine fluoride fibres having an internal diameter of 1 mm. The fibres are heat-sealed at 2 cm intervals, and the samples are placed into tissue culture medium and incubated for 24 to 48 hours prior to implantation. On the day of implantation, samples of each tumour cell line preparation are quantitated for viable cell mass by a stable endpoint MTT assay so that the time zero cell mass is known. Each mouse receives 3 i.p. implants representing 3 of the tumour cell lines, and 3 s.c. implants of the same 3 tumour cell lines. Mice are treated with experimental agents, which have been solubilized using 10% DMSO in saline and tween 80 (0.05%), on day 3 or 4 following fibre implantation and continuing daily for 4 days. Each agent is administered by i.p. injection at 2 dose levels. The dose levels are determined from the single dose i.p. maximum tolerated dose (MTD) for each test agent; the high dose being the MTD $\times$ 0.375 and the low dose being the MTD $\times$ 0.25. The fibres are collected from the mice on the day following the fourth treatment and viable cells estimated by the MTT assay. A 50% or greater reduction in percent net growth in the treated samples compared to the vehicle control samples is considered a positive result. A total of 48 fibres is treated in a standard experiment (12 cell lines $\times$ 2 implant sites $\times$ 2 dose levels). Each treated fibre exhibiting at least a 50% net reduction in cell growth is assigned an arbitrary score of 2 points, and the number of fibres reaching this level in the i.p. and s.c. compartments are recorded separately. Any cell lines in which cell kill is observed are also recorded.

Criteria were initially established for compound activity using a training set of 80 randomly selected compounds which were evaluated in both the HF and xenograft assays. The goal of the training set was to define a scoring system which allowed bias in favour of detecting all compounds with xenograft activity, defined as ≤40% T/C in at least one tumour xenograft. Criteria for activity in the HF assay which accomplished this goal were thus: 20 or more total points (any combination of points in the i.p. and s.c. fibres), 8 or more points in s.c. fibres, or an observation of cell kill in any fibre in either the i.p. or s.c. compartment.

The NCI in vitro anti-cancer drug screen has been described in detail previously (Alley et al, 1988; Monks et al, 1991). It utilizes 60 different human tumour cell lines representing leukaemia, melanoma, lung, colon, brain, ovary, breast, prostate and kidney cancers. For a typical screening experiment, cells are inoculated into 96-well microtitre plates and allowed to incubate for 24 hours. Experimental drugs are solubilized in dimethyl sulfoxide and stored frozen prior to use. At the time of drug addition, an aliquot of frozen concentrate is thawed and diluted with complete medium. 4,10-fold or 1/2 log additional serial dilutions are made to provide a total of 5 drug concentrations plus control. Following drug addition, the plates are incubated for an additional 48 hours. Sulforhodamine B solution is added to each well, and bound stain is solubilized and the absorbance read. The percentage growth is calculated at each of the drug concentration levels. 3 dose response parameters are calculated for each experimental agent: growth inhibition of 50% ($GI_{50}$), total growth inhibition (TGI), and the concentration of drug resulting in a 50% reduction in measured protein ($LC_{50}$).

### Structural characteristics

Chem-X, a product of Chemical Design, Ltd (Oxfordshire, UK), was used to quantitate aspects of structural characteristics such as the number of hydrogen bonds, hydrogen bond donors and acceptors. Molecular weights were taken as calculated from molecular formulas from the DTP Drug Information System.

### Statistical tests

$\chi^2$ analyses were conducted to examine the effect of various factors on in vivo outcomes; where insufficient data were available for a $\chi^2$ analysis, the Fisher's exact test was employed. The Spearman rank correlation coefficient was used to correlate in vivo activity with clinical responses; this statistic is defined as the more familiar Pearson correlation of the data, after the data values for each measure of activity have been replaced by their respective ranks. The Spearman rank correlation is more appropriate than the Pearson correlation in situations where the data are not normally distributed. A Student's *t*-test was used in comparing mean MW and means for hydrogen-bonding characteristics. Statistical significance was set at the 99% confidence level ($P < 0.01$).

### RESULTS

### Indicators of clinical activity

For 39 clinical agents, relationships between xenograft response levels, averaged by histology, and the phase II response rates were investigated. The Spearman rank correlation coefficients (r) for all histologies are plotted in Figure 1. Only non-small cell lung (NSCL) xenografts were predictive of clinical activity in the same histology (r = 0.814, $P = 0.004$). Breast xenograft models were the most useful for predicting clinical response against any disease, correlating with clinical activity against NSCL (r = 0.565, $P = 0.008$), melanoma (r = 0.540, $P = 0.007$) and ovarian (r = 0.611, $P = 0.003$) cancer, but interestingly, not with clinical breast cancer. Activity in colon xenografts predicted for clinical melanoma response (r = 0.532, $P = 0.005$). There were no other correlations that met the criteria for statistical significance. The number of xenograft models with a T/C ≤40% or ILS ≥25% was compared with the presence of clinical activity. Clinical activity was found in only 2/6 agents (33%) with activity in fewer than one-third or more of tested xenograft models, but was found in 21/33 agents (64%) with activity in one-third or more of tested xenograft models (Figure 2A); undoubtedly due to the small number of agents, this difference is not statistically significant ($P = 0.14$). The comparison was also repeated using a more stringent definition of clinical activity, demanding that agents tested in multiple disease demonstrate response in at least 2 diseases. Applying this more strigent definition, clinical activity was found in 0/6 agents (0%) which had activity in fewer than one-third of tested pre-clinical xenograft models, whereas 15/33 agents (45%) with activity in one-third or more of tested pre-clinical xenograft models had clinical activity ($P = 0.04$) (Figure 2B). The analyses were repeated, increasing the threshold for xenograft activity to a T/C ≤10% or ILS ≥50%, a comparison which yielded no useful correlations between overall xenograft activity and either definition of clinical activity.

### Indicators of xenograft activity

To assess how HF activity might be related to predicting activity in some xenograft model, we considered the likelihood of responses in xenograft models as a function of HF activity. Table 2A shows

 © 2001 Cancer Research Campaign

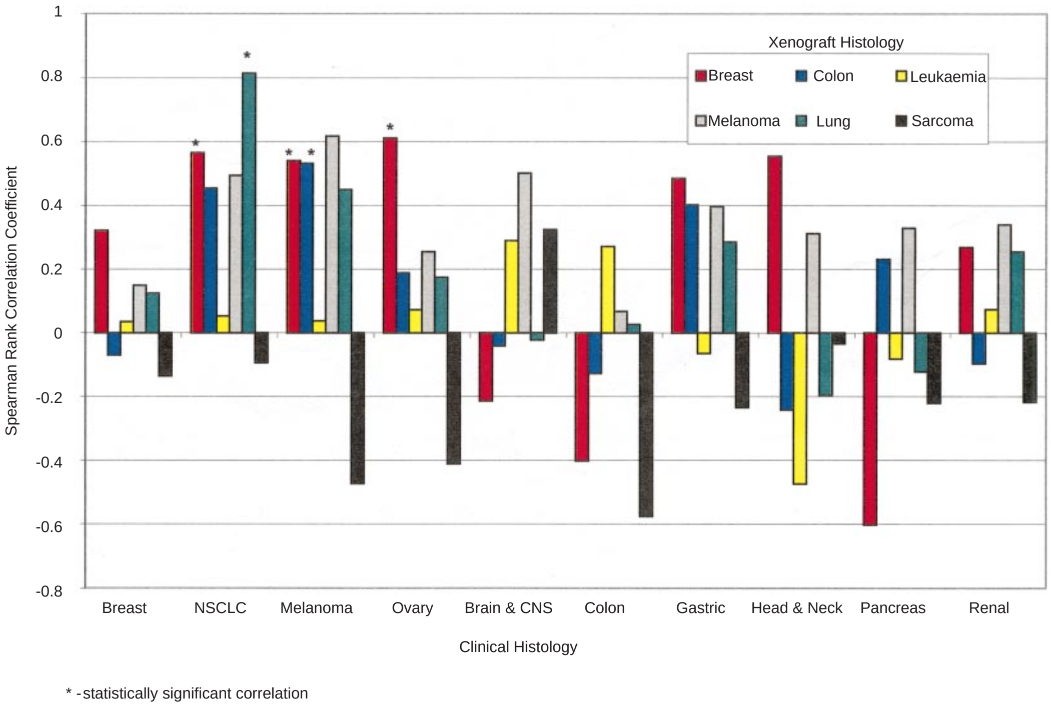

**Figure 1** In vivo activity and clinical activity by disease type The median number of agents per correlation was 12 (range 2–27). For the statistically significant correlations, the median was 21 (range 10–26). The number of xenograft systems per histology ranged between 7 and 9 for all histologies except for sarcoma which included only one xenograft model. * – statistically significant correlation

**Table 2A** IP HF activity vs xenograft activity (at least 4 tumours tested)

| | % of xenograft models active | | | |
|---|---|---|---|---|
| **Responsive IP fibres** | **<33%** | **≥33%** | **Total** | **% active in 33% of xenografts** |
| 0–6 fibres | 179 | 6 | 185 | 3% |
| ≥7 fibers | 57 | 14 | 71 | 20% |
| Total | 236 | 20 | 256 | 8% |
| | | | $\chi^2$ = 19.3, $P$ < 0.0001 | |

**Table 2B** IP HF activity vs activity in IP xenograft models

| Responsive IP fibres | Agents active in IP xenograft | Agents inactive in IP xenograft | Total | % active |
|---|---|---|---|---|
| 0–3 fibres | 32 | 231 | 263 | 12% |
| 4–6 fibres | 23 | 85 | 108 | 21% |
| 7–9 fibres | 16 | 30 | 46 | 35% |
| ≥10 fibres | 19 | 23 | 42 | 45% |
| Total | 90 | 369 | 459 | 20% |
| | | | $\chi^2$ = 33.7, $P$ < 0.0001 | |

Compounds were studied in a median of one ip xenograft model, with some compounds being tested in as many as 8 models.

**Table 2C** IP HF activity vs activity in any xenograft model

| Responsive IP fibres | Agents active in any xenograft | Agents inactive in all xenograft | Total | % active |
|---|---|---|---|---|
| 0–3 fibres | 84 | 212 | 296 | 28% |
| 4–6 fibres | 43 | 84 | 127 | 34% |
| 7–9 fibres | 26 | 31 | 57 | 46% |
| ≥10 fibres | 36 | 21 | 57 | 63% |
| Total | 189 | 348 | 537 | 35% |
| | | | $\chi^2$ = 28.4, $P$ < 0.0001 | |

that using compounds with studies in at least 4 different tumour types, there exists a strong correlation ($P$ < 0.0001) between activity in HF placed in the peritoneal compartment and ultimate xenograft activity, so that compounds with activity in >6 peritoneal fibres had a 20% likelihood of subsequent xenograft activity while compounds with activity in ≤6 fibres had a 3% rate of xenograft activity. One criticism of this correlation is that it would be relevant only to intraperitoneal xenografts. Table 2B does in fact show that activity in ≥10 peritoneal fibres does, perhaps not surprisingly, show activity in 45% of peritoneal xenografts. However, Table 2C reinforces the correlation of activity in peritoneal fibres to activity in any xenograft model, including subcutaneous models. Note that for this comparison, if the threshold for HF activity is raised to more than 6 fibres, at least two-thirds of the xenograft active compounds become false negatives. Interestingly, activity in subcutaneously placed fibres did not correlate with likely activity in either peritoneal or subcutaneous xenografts (data not shown). Since HF response did not account for all of the xenograft active agents, the nature of the compounds and test conditions for these agents were examined. In all of these compounds, the xenograft activity was obtained using a route, schedule or other experimental condition not available or not routinely used in the HF assay (data not shown); in the case of some slower growing solid tumours, for example, activity was obtained using Q4Dx3 or Q7Dx3 schedules which are not available in the standard 4-day HF assay.

A second general indicator of likely xenograft activity actually emerges from consideration of in vitro screening data. Tables 3A

© 2001 Cancer Research Campaign 

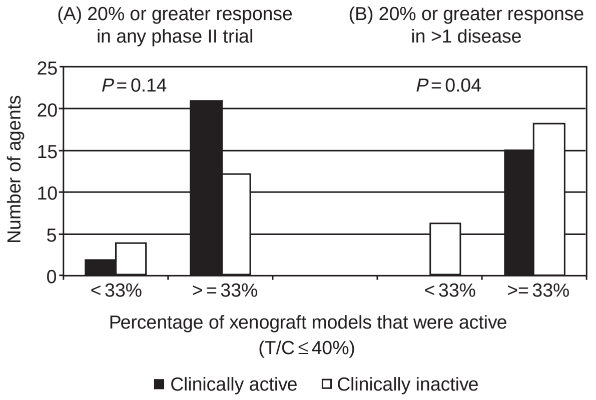

**Figure 2** Overall xenograft activity & clinical activity.
Agents were tested in a median of 12 xenografts (range 2–45) and a median of 4 clinical diseases (range 1–24). The 33% cutoff was chosen retrospectively to provide a useful benchmark which minimized false negatives

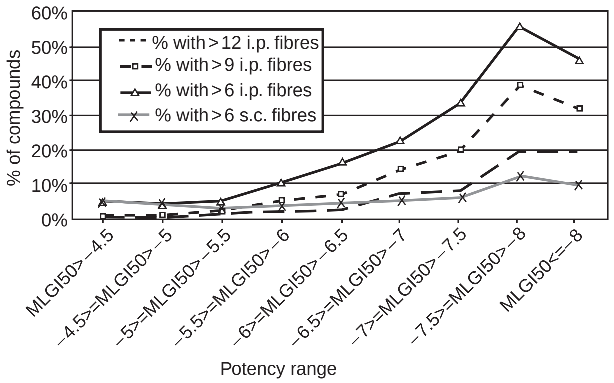

**Figure 3** Mean log of $GI_{50}$ versus percentage of compounds with hollow fiber response in at least *n* fibers and 3B demonstrate that compounds with evidence of selective activity for successively greater numbers of lung or breast carcinoma cell lines had successively increased likelihood of demonstrating activity in lung or breast xenograft models. Compounds with evidence of in vitro selectivity in 6 or more lung cell lines exhibited activity in 33% (compared to 17.5% overall) of corresponding xenografts. Compounds with evidence of in vitro selectivity in breast cell lines were even more likely (44%) to be active in the corresponding xenografts (compared to 21% overall). When this effort was extended to other histologies, no other significant correlations emerged (data not shown).

### Indicators of HF activity

Given that HF response can, to a certain extent, predict some level of xenograft activity, it becomes of interest to enquire if predictors of activity in the HF model can be gleaned from in vitro screening data or structural properties of the molecules. Table 4 demonstrates that in the case of breast, lung, ovarian, CNS and melanoma cell lines, selective activity in the particular in vitro panel does show a trend toward correlating with emergence of activity in the corresponding cell types in the HF panel. For example, 48% of the agents that were selective for breast cell line activity in vitro also showed activity (growth inhibition of 50%) in the breast carcinoma HF, compared to 39% of compounds selected for study in hollow fibres for any reason. Colon carcinoma cell line activity did not correlate with HF colon cancer cell line activity.

Greater potency in the 60 cell line assay, as indicated by the $GI_{50}$, correlates exceptionally well with increasing activity in the HF model with peritoneal fibres (Figure 3) ($P$ < 0.0001). For example, 37% of compounds with a $\log_{10}$ $GI_{50}$ of at least -7.5 demonstrate activity in at least 10 out of 24 i.p. fibres, whereas the fraction of all compounds with this level of activity in the HF assay is only 6%. Similar analyses were carried out utilizing activity in s.c. fibres and i.p. plus s.c. fibres. As with the comparison of HF-to-xenografts, these analyses showed that activity in the s.c. fibres did not correlate with in vitro 60 cell line potency, and that activity in the i.p. plus s.c. fibres was less correlative than the i.p. fibre response alone (data not shown).

The mean $GI_{50}$ for only the 12 cell lines used in the HF assay was also examined to determine if this measure was more correlative than the average potency in all 60 cell lines. Although 12 cell line in vitro potency still correlates positively with HF activity, the correlation is not as striking as that obtained using the 60 cell line $GI_{50}$ (data not shown).

'Differential' activity of a compound between distinct cell types in the in vitro screen may be an indication of a basis for expecting selective anti-proliferative effect in vivo and ultimately in the

**Table 3A** Analysis of in vitro lung histology response vs lung xenograft response

| No. lung lines with $GI_{50}$ < mean $GI_{50}$ | Lung xenograft inactive | Lung xenograft active | Total tested | % active | $\chi^2$ | *P* |
|---|---|---|---|---|---|---|
| 3 | 299 | 79 | 378 | 21% | 9.99 | 0.00158 |
| 4 | 206 | 70 | 276 | 25% | 24.1 | <0.0001 |
| 5 | 133 | 56 | 189 | 30% | 29.6 | <0.0001 |
| 6 | 78 | 38 | 116 | 33% | 23.8 | <0.0001 |

**Table 3B** Analysis of in vitro breast histology response vs breast xenograft response

| No. breast lines with $GI_{50}$ < mean $GI_{50}$ | Breast xenograft inactive | Breast xenograft active | Total tested | % active | $\chi^2$ | *P* |
|---|---|---|---|---|---|---|
| 3 | 189 | 53 | 242 | 22% | 0.665 | 0.415 |
| 4 | 159 | 46 | 205 | 22% | 0.884 | 0.347 |
| 5 | 103 | 38 | 141 | 27% | 5.62 | 0.0180 |
| 6 | 34 | 27 | 61 | 44% | 25.0 | <0.0001 |

 © 2001 Cancer Research Campaign

**Table 4** Histological comparison between 60 cell line screen and hollow fibre assay

| HF disease type | I. | II. | $\chi^2$ | *P* |
|---|---|---|---|---|
| Breast | 48% | 39% | 14.9 | 1.11E–04 |
| Colon | 26% | 27% | 0.182 | 0.670 |
| Lung | 65% | 59% | 10.7 | 0.00106 |
| Ovarian | 55% | 40% | 14.5 | 1.42E–04 |
| CNS | 58% | 47% | 15.9 | <0.0001 |
| Melanoma | 30% | 25% | 8.85 | 0.00293 |

I. This column shows the activity rate for the HF tumour type where 4 or more cell lines in the same 60 cell line disease panel were responsive.
II. This column shows the overall activity rate for the HF tumour type for all compounds tested.

**Table 5** Differential activity and HF activity

| Δ | Responsive IP fibres 0 | 1–3 | 4–6 | 7–9 | ≥10 | Total | % with 4 or more fibres |
|---|---|---|---|---|---|---|---|
| Δ≤.5 | 34 | 76 | 33 | 20 | 36 | 199 | 45% |
| 0.5<Δ≤1 | 138 | 427 | 154 | 46 | 50 | 815 | 31% |
| 1<Δ≤1.5 | 179 | 389 | 138 | 38 | 42 | 786 | 28% |
| 1.5<Δ≤2 | 77 | 150 | 55 | 21 | 18 | 321 | 29% |
| Δ>2 | 39 | 97 | 38 | 6 | 3 | 183 | 26% |
| Total | 467 | 1139 | 418 | 131 | 149 | 2304 | 30% |

$\chi^2$ = 79.4, *P* < 0.0001

**Table 6** Molecular weight and HF activity

| MW | Responsive IP fibres 0 | 1–3 | 4–6 | 7–9 | ≥10 | Total | % with 4 or more fibres |
|---|---|---|---|---|---|---|---|
| MW≤250 | 47 | 102 | 27 | 11 | 2 | 189 | 21% |
| 250<MW≤500 | 330 | 815 | 311 | 78 | 103 | 1637 | 30% |
| 500<MW≤750 | 68 | 175 | 65 | 27 | 30 | 365 | 33% |
| 750<MW≤1000 | 14 | 25 | 10 | 13 | 7 | 69 | 43% |
| MW>1000 | 8 | 22 | 4 | 1 | 7 | 42 | 29% |
| Total | 467 | 1139 | 417 | 130 | 149 | 2302 | 30% |

$\chi^2$ = 55.3, *P* < 0.0001

**Table 7** Hydrogen bonding sites and HF activity

| Potential H bond sites | Responsive IP fibres 0 | 1–3 | 4–6 | 7–9 | ≥10 | Total | % with 4 or more fibres |
|---|---|---|---|---|---|---|---|
| 0 to 3 | 110 | 233 | 70 | 21 | 8 | 442 | 22% |
| 4 to 6 | 226 | 577 | 223 | 53 | 58 | 1137 | 29% |
| 7 to 10 | 105 | 250 | 97 | 33 | 59 | 544 | 35% |
| 11 to 15 | 16 | 51 | 21 | 17 | 13 | 118 | 43% |
| ≥16 | 10 | 28 | 7 | 7 | 11 | 63 | 40% |
| Total | 467 | 1139 | 418 | 131 | 149 | 2304 | 30% |

$\chi^2$ = 88.3, *P* < 0.0001

**Table 8** Molecular weight and HF activity (independent of hydrogen bonding sites)

| MW | Responsive IP fibres (4–6 hydrogen bonding sites) 0 | 1–3 | 4–6 | ≥7 | Total |
|---|---|---|---|---|---|
| MW≤250 | 24 | 41 | 15 | 4 | 84 |
| 250<MW≤500 | 178 | 488 | 186 | 101 | 953 |
| MW>500 | 27 | 48 | 19 | 6 | 100 |
| Total | 229 | 577 | 220 | 111 | 1137 |

$\chi^2$ = 11.0, *P* = 0.0895

**Table 9** Hydrogen bonding and HF activity (independent of MW)

| Potential H-bond sites | Responsive IP fibres (250 < MW ≤500) 0 | 1–3 | 4–6 | 7–9 | ≥ 10 | Total |
|---|---|---|---|---|---|---|
| 0–3 | 86 | 163 | 55 | 14 | 8 | 326 |
| 4–6 | 175 | 488 | 188 | 45 | 56 | 952 |
| ≥7 | 69 | 164 | 68 | 19 | 39 | 359 |
| Total | 330 | 815 | 311 | 78 | 103 | 1534 |

$\chi^2$ = 35.8, *P* < 0.0001

clinic. Indeed, the potential for this to emerge was a key rationale for embarking on the 60 cell line screening paradigm (Alley et al, 1988). The difference in $GI_{50}$ for any cell line and the average $GI_{50}$ for all 60 cell lines was computed for the compounds studied in the peritoneal fibres. The largest differential or 'Δ' over the 60 cell lines is recorded by cell type and value for each compound. Table 5 shows that increasing Δ actually correlates negatively with increasing likelihood of observance of activity in the HF model ($P$ < 0.0001). For example, the percentage of compounds which respond in at least 4 i.p. fibres is greater with a Δ of ≤0.5 (45%) than the percentage with Δ > 2 (26%). The same trend is displayed when comparing Δ with xenograft activity, but the correlation is not statistically significant (data not shown).

The effect of molecular weight and the number of potential hydrogen bonds in the compound structure on activity in the HF assay were also examined. Compounds in low MW ranges (160 to 480) are generally considered to be more 'drug-like' than those in higher ranges (Ghose et al, 1999). Interestingly, the number of responsive i.p. fibres generally increases with increasing MW up to a MW of 1000 (Table 6). For MW greater than 1000 activity appears to decrease, but there are too few compounds of this size level to generalize. In molecules with 7 to 10 potential hydrogen bonds, 35% of compounds responded in a least 6 i.p. fibres versus 30% overall ($P$ < 0.0001) (Table 7). This percentage increased to 43% in compounds with 11 or more potential hydrogen bonds. Since, however, the number of heteroatoms contributes to both the number of hydrogen bonds and MW, interdependence of MW and hydrogen bond counts was examined and the results shown in Table 8. Using the constant hydrogen bond range of 4 to 6, the correlation between MW and HF activity was no longer statistically significant ($P$ = 0.0895). However, using the constant MW range of 250 to 500, the hydrogen bond parameter, now independent of the effect of MW, still correlated strongly with HF response ($P$ < 0.0001) (Table 9). Neither MW or the number of hydrogen bonds had any influence on activity in the s.c. fibres. The

*© 2001 Cancer Research Campaign*

relationship between these physiological properties and xenograft activity had also been examined; although the same conclusions could be drawn, the correlations were not as strong (data not shown).

According to the Lipinski Rule to Five (Lipinski et al, 1997), compounds with more than 5 hydrogen bond donors (HBD) or more than 10 hydrogen bond acceptors (HBA) are more likely to have poor oral absorption. Among the compounds tested in the HF assay, 95% had less than 5 HBD and 95% less than 10 HBA; since the percentages of compounds with favourable HBD and HBA counts was so great, an analysis of the effect of these parameters was not performed. In fact, these parameters were identical to the compounds entering the in vitro screening system.

Unfortunately, only about 75% of compounds selected for the HF assay are tested in that system, primarily due to problems with insufficient supply of compound. The 25% of selected but not tested compounds represent a potential source of bias in these analyses, particularly as the compounds for which it is not possible to obtain the quantities necessary for in vitro testing might represent greater structural complexity. In fact, the mean MW of non-tested compounds was 485 (vs 420 for tested compounds), with only approximately 1.3 additional H bond sites per molecule (data not shown).

### DISCUSSION

The analysis of xenograft versus clinical results illustrates that a histology to histology comparison of these models to activity in the clinic cannot be reliably discerned for these ‘empirically’ selected compounds acting against non-molecularly characterized tumours. Although, with the exception of lung, histological matches were not found between in vivo models and clinical response, activity in multiple xenograft models does appear to predict for some degree of clinical activity. Interestingly, increasing the activity threshold to a T/C of ≤10 did not increase the likelihood of a positive clinical outcome, indicating that this activity threshold is probably too stringent. Requiring greater clinical activity (activity in 2 or more diseases) does improve the correlation between xenograft activity and clinical activity. Although the more stringent definition of clinical activity may not be suitable for making decisions on whether to advance agents from phase II to phase III trials, as a purely statistical exercise, it does support the conclusion that activity in multiple xenograft models is a useful predictor of clinical activity.

In contrast to results achieved with the DTP xenograft system, Fiebig (Scholz et al, 1990) has developed xenograft tumours which retain characteristics similar to the clinical specimens, having been grown from slow-growing and well-differentiated cell lines. Fiebig reports that when treating these xenograft tumours with the same standard agents as were utilized in the clinic for the corresponding patient tumours, the response of these xenografts in comparison to patient tumours was 90% (19/21) for sensitive and 97% (57/59) for non-responding tumours respectively. While this degree of correspondence between clinically used agent activity in xenografts and the clinic is gratifying, it is uncertain how to translate that experience to the evaluation of new agents with no prior defined clinical activity. As the clinically used standard agents have in all cases proven active in many xenograft models, this result at one level might be in accord with our finding that the number of xenografts in which an agent is active correlates with likely activity in the clinical setting.

The results presented in Figures 1 and 2 may be taken to argue against the use of activity in an empirically selected xenograft model to predict activity in the same histologic type of cancer in the clinic, and indeed that result has influenced the current philosophy underlying NCI’s drug discovery and development programme (Sausville and Feigal, 1999). Nonetheless, definition of an active agent in xenografts would allow optimization of schedule, assessment of molecular target endpoints, and increase the probability of clinical activity. It is therefore beneficial to have the ability to select agents with a likelihood of xenograft activity using a rapid and inexpensive test. The HF assay was developed to serve as a discriminator for compounds emerging from an empirical in vitro cell line screen. Our analyses indicate that greater levels of response in the i.p. fibres correlate with greater likelihood of xenograft activity. The same cannot be stated for responses in the s.c. fibres. Double (Phillips et al, 1998) reports that the NCI protocol for HF does not allow sufficient time for angiogenesis to occur, and hypothesizes that s.c. response may be underestimated with NCI’s protocol. Folkman (Hahnfeldt et al, 1999), however, claims that a tumour does not require vessels until it is 3–4 mm in diameter; the polyvinylidene fibres are only 1 mm. Subcutaneous response is dependent on extravascular drug concentration, so the lack of correlation between s.c. fibres response and xenograft activity may be speculatively related to factors such as dose, route, schedule, hydrostatic pressure and hydration state of the host.

As with xenograft to clinical comparisons, activity against a single specific HF histology was generally not useful for predicting xenograft activity in the same histology. The lack of histologic correlations in this case can perhaps be explained by the minimal number (2 of each histology) in the HF assay. In contrast, most panels in the 60 cell line in vitro assay consist of a minimum of 6 different cell types, so cell panel specificity is more readily defined. Note that the correlations between in vitro and either HF or xenografts are only apparent when 4 or more cell lines respond.

The examination of the in vitro screening data indicate strong histologic correlations with HF activity for all panels but colon, while indications of differential activity (high ‘Δ’), did not correlate with HF activity. This observation combined with that of the correlation of HF response with increasing in vitro potency may indicate that the standard HF assay is sensitive to strongly cytotoxic as opposed to ‘differentially’ acting agents. It can also be taken to indicate that if differential activity is truly suggested from in vitro results, a HF experiment addressing that particular set of cell types may need to be designed or specific target manipulation should be considered.

Our analysis of MW and hydrogen-bonding factors revealed a correlation between increasing MW, as well as greater hydrogen-bonding sites and HF activity. This suggests that in choosing test agents for further development, a compromise should be made between low MW for drug development purposes and the number of hydrogen-bonding sites for efficacy. HBA and HBD counts were not revealing, as 95% of the input to the in vitro screen was in the favourable range for these factors.

These results may be considered to define a ‘road-map’ for charting the transition of a compound from an in vitro screening result to a clinical candidate. If the development strategy of a compound is to remain empiric, consideration of the heterogeneous nature of in vivo model to clinical correlation might require strategies to define the likelihood of clinical activity, and again overall potency and activity in a large number of HF fibres might allow the best delineation of compounds to consider for further

© 2001 Cancer Research Campaign development. It could be argued from this experience that compounds with an anti-proliferative effect at $10^{-6}$ M have a relatively higher likelihood of affecting all growth in a responsive tumour. Compounds of the future will likely be advanced to clinical testing with an eye toward addressing a defined molecular abnormality or target in the tumour cell. Greater numbers ($n \geq 4$) of redundant cell types expressing the target, whether in an in vitro assay such as the 60 cell line screen, or an in vivo assay such as the HF assay, might be expected to have concordant effects in xenografts bearing the same target. Finally, as efforts are consolidated to derive engineered animal strains to provide models of tumour biology and pathophysiology (http://www.nci.nih.gov/dcb/odhome.htm#MOUSE), consideration of the 'baseline' experience of an empirically oriented drug discovery program might usefully benchmark the types of compounds suitable for advancement to such models.

### ACKNOWLEDGEMENTS

Laboratory studies supporting the analyses reported herein were conducted in whole or in part with Federal funds from the National Cancer Institute, National Institutes of Health, under Contracts NO1-CO-5600 and NO-1-CM-47000. Mention of trade names or commercial products in this publication does not imply endorsement by the US Government.

### REFERENCES

Alley MC, Scudiero DA, Monks A, Hursey ML, Czerwinski MJ, Fine DL, Abbott BJ, Mayo JG, Shoemaker RH and Boyd MR (1988) Feasibility of drug screening with panels of human tumor cell lines using a microculture tetrazolium assay. *Cancer Res* **48**: 589–601

DeVita VT, Hellmann S and Rosenberg SA (1982) *Cancer Principles and Practice of Oncology*, Lippincott-Raven: Philadelphia

DeVita VT, Hellmann S and Rosenberg SA (1985) *Cancer Principles and Practice of Oncology*, Lippincott-Raven: Philadelphia

DeVita VT, Hellmann S and Rosenberg SA (1989) *Cancer Principles and Practice of Oncology*, Lippincott-Raven: Philadelphia

DeVita VT, Hellmann S and Rosenberg SA (1993) *Cancer Principles and Practice of Oncology*, Lippincott-Raven: Philadelphia

DeVita VT, Hellmann S and Rosenberg SA (1997) *Cancer Principles and Practice of Oncology*, Lippincott-Raven: Philadelphia

Dykes DJ, Abbott BJ, Mayo JG, Harrison Jr SD, Laster Jr WR, Simpson-Herren L and Griswold Jr DP (1992) Development of human tumor xenograft models for in vivo evaluation of new antitumor drugs. *Contrib Oncol* **42**: 1–22

Gellhorn A and Hirschberg E (1955) Investigation of diverse systems for cancer chemotherapy screening. *Cancer Res Suppl* **3**: 1–125

Ghose AK, Viswanadhan VN and Wendoloski JJ (1999) A knowledge-based approach in designing combinatorial or medicinal chemistry libraries for drug discovery. 1. A qualitative and quantitative characterization of known drug databases. *J Comb Chem* **1**: 55–68

Hahnfeldt P, Panigrahy D, Folkman J and Hlatky L (1999) Tumor development under angiogenic signaling: a dynamical theory of tumor growth, treatment response, and postvasular dormancy. *Cancer Res* **59**: 4770–4775

Hollingshead M, Plowman J, Alley M, Mayo J and Sausville E (1999) The hollow fiber assay. In: *Contributions to Oncology, Volume 54: Relevance of Tumor Models for Anticancer Drug Development*, Fiebig H and Burger AM (eds) pp 109–120. Karger: Freiburg

Lipinski CA, Lombardo F, Dominy BW and Feeney PJ (1997) Experimental and computational approaches to estimate solubility and permeability in drug discovery and development settings. Adv *Drug Delivery Rev* **23**: 3–25

Monks A, Scudiero D, Skehan P, Shoemaker R, Paull K, Vistica D, Hose C, Langley J, Cronise P, Vaigro-Wolff A, Gray-Goodrich M, Campbell H, Mayo J and Boyd M (1991) Feasibility of a high-flux anticancer drug screen using a diverse panel of cultured human tumor cell lines. *J Natl Cancer Inst* **83**: 757–766

Paull KD, Shoemaker RH, Hodes L, Monks A, Scudiero DA, Rubinstein L, Plowman J and Boyd MR (1989) Display and analysis of patterns of differential activity of drugs against human tumor cell lines: development of the mean graph and COMPARE algorithm. *J Natl Cancer Inst* **81**: 1088–1092

Paull KD, Hamel E and Malspeis L (1995) Prediction of biochemical mechanism of action from the in vitro antitumor screen of the National Cancer Institute. In: *Cancer Chemotherapeutic Agents*, Foye WO (ed) pp 9–45. Americal Chemical Society: Washington, DC

Phillips RM, Pearce J, Loadman PM, Bibby MC, Cooper PA, Swaine DJ and Double JA (1998) Angiogenesis in the hollow fiber tumor model influences drug delivery to tumor cells: implications for anticancer drug screening programs. *Cancer Res* **58**: 5263–5266

Plowman J, Dykes DJ, Hollingshead M, Simpson-Herren L and Alley MC (1997) Human tumor xenograft models. In: *Anticancer Drug Development Guide: Preclinical Screening, Clinical Trials, and Approval*, Teicher B (ed) pp 101–125. Humana Press: Totowa, NJ

Sausville EA and Feigal E (1999) Evolving approaches to cancer drug discovery and development at the National Cancer Institute, USA. *Annals Oncol* **10**: 1287–1291

Scholz CC, Berger DP, Winterhalter BR, Henss H and Fiebig HH (1990) Correlation of drug response in patients and in the clonogenic assay with solid human tumour xenografts. *Eur J Cancer* **26**: 901–905

Venditti JM (1981) Preclinical drug development: rationale and methods. *Seminars Oncol* **8**: 349–361

Venditti JM, Wesley RA and Plowman J (1984) Current NCI preclinical antitumor screening in vivo: results of tumor panel screening, 1976–1982, and future directions. *Adv Pharmacol Chemother* **20**: 1–19

Zubrod CG, Schepartz S, Leiter J, Endicott KM, Carrese LM and Baker CG (1966) The chemotherapy program of the National Cancer Institute: history, analysis and plans. *Cancer Chemother Rep* **50**: 349–540

*© 2001 Cancer Research Campaign*
